# Knockdown of long non-coding RNA XIST increases blood–tumor barrier permeability and inhibits glioma angiogenesis by targeting miR-137

**DOI:** 10.1038/oncsis.2017.7

**Published:** 2017-03-13

**Authors:** H Yu, Y Xue, P Wang, X Liu, J Ma, J Zheng, Z Li, Z Li, H Cai, Y Liu

**Affiliations:** 1 Department of Neurosurgery, Shengjing Hospital of China Medical University, Shenyang, People’s Republic of China; 2 Liaoning Research Center for Translational Medicine in Nervous System Disease, Shenyang, People’s Republic of China; 3 Department of Neurobiology, College of Basic Medicine, China Medical University, Shenyang, People’s Republic of China; 4 Key Laboratory of Cell Biology, Ministry of Public Health of China, and Key Laboratory of Medical Cell Biology, Ministry of Education of China, China Medical University, Shenyang, People’s Republic of China

## Abstract

Antiangiogenic therapy plays a significant role in combined glioma treatment. However, poor permeability of the blood–tumor barrier (BTB) limits the transport of chemotherapeutic agents, including antiangiogenic drugs, into tumor tissues. Long non-coding RNAs (lncRNAs) have been implicated in various diseases, especially malignant tumors. The present study found that lncRNA X-inactive-specific transcript (XIST) was upregulated in endothelial cells that were obtained in a BTB model *in vitro*. XIST knockdown increased BTB permeability and inhibited glioma angiogenesis. The analysis of the mechanism of action revealed that the reduction of XIST inhibited the expression of the transcription factor forkhead box C1 (FOXC1) and zonula occludens 2 (ZO-2) by upregulating miR-137. FOXC1 decreased BTB permeability by increasing the promoter activity and expression of ZO-1 and occludin, and promoted glioma angiogenesis by increasing the promoter activity and expression of chemokine (C–X–C motif) receptor 7b (CXCR7). Overall, the present study demonstrates that XIST plays a pivotal role in BTB permeability and glioma angiogenesis, and the inhibition of XIST may be a potential target for the clinical management of glioma.

## Introduction

Glioblastoma (GBM) is the most lethal brain tumor, which is generally incurable. Surgical resection combined with postoperative radiochemotherapy is a common treatment strategy for the tumors, but the median survival time for patients with GBM remains disheartening.^1^ GBM is characterized by the elevated expression of proangiogenic factors and heightened tumor angiogenesis,^2^ which makes antiangiogenic therapy promising for GBM treatment. The blood–tumor barrier (BTB) mainly consists of highly specialized endothelial cells (ECs). It restricts the delivery of most antitumor drugs to the brain tumor,^3^ which ultimately mitigates the effects of chemotherapy. Therefore, increasing BTB permeability in parallel with inhibiting glioma angiogenesis may be a more efficient treatment strategy for glioma.

Similar to the brain–blood barrier, tight junction-related proteins and adhesion junctions are the main molecular components of the BTB. Tight junction-related proteins include zonula occludens (ZO) proteins, occludin, claudins and junction adhesion molecular.^4^ Targeting intercellular tight junctions in glioma endothelial cells (GECs) may facilitate drug delivery through the paracellular pathway.

Growing evidence indicates that long non-coding RNAs (lncRNAs) are involved in the progression of various human tumors.^5, 6, 7^ In our previous study, we found that lncRNA X-inactive-specific transcript (XIST) was upregulated in GBM patients and glioma stem cells. XIST knockdown inhibited the malignant behavior of glioma stem cells.^8^ To date, the role of XIST in BTB permeability and glioma angiogenesis remains unclear.

miR-137 is downregulated in glioma samples and cell lines. The overexpression of miR-137 inhibits the proliferation, invasion and angiogenesis of glioma cells.^9, 10, 11, 12^ miR-137 is also involved in regulating the behavior of ECs. The ectopic expression of miR-137 suppresses the viability and migration of human umbilical vein endothelial cells.^13^ Using Starbase 2.0 (http://starbase.sysu.edu.cn/; accessed 22 December 2016), we found that XIST has a putative miR-137-binding site, indicating that miR-137 may participate in the XIST regulatory network.

Forkhead box C1 (FOXC1) is a member of the forkhead box family and regulates the behavior of gastric cancer,^14^ breast cancer^15^ and tongue cancer.^16^ In the central nervous system, FOXC1 mutations lead to small-vessel disease in the mouse brain.^17^ FOXC1 deficiency causes the abnormal vascular formation of the mouse telencephalon.^18^ However, whether FOXC1 is involved in regulating of BTB permeability and angiogenesis remains unknown. Bioinformatics tools show that FOXC1 is predicted to be a target of miR-137, suggesting that miR-137 may regulate the expression and function of FOXC1.

The present study investigated the expression of XIST, miR-137 and FOXC1 in GECs, and their role in BTB permeability and glioma angiogenesis. We sought to clarify the crosstalk between XIST, miR-137 and FOXC1.

## Results

### XIST was upregulated in GECs, and XIST knockdown increased BTB permeability and inhibited glioma angiogenesis

**Figure 1:**
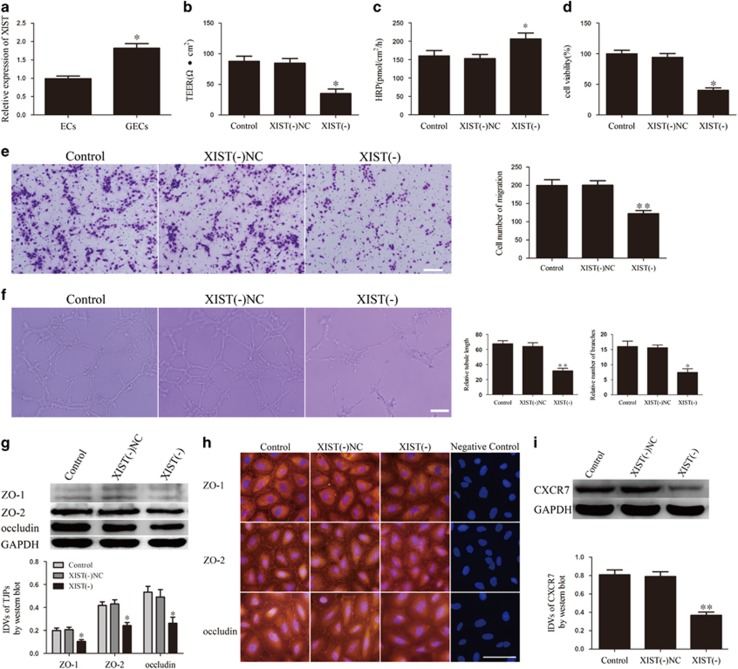
XIST expression in GECs and XIST regulated BTB permeability, glioma angiogenesis, tight junction-related proteins and CXCR7 expression. (**a**) Relative XIST expression in ECs and GECs by real-time qPCR. Data represent mean±s.d. (*n*=5, each). *P<0.05 vs ECs group. (**b**) Effect of XIST knockdown on TEER in BTB model *in vitro*. (**c**) Effect of XIST knockdown on HRP flux in BTB model *in vitro*. (**d**) Effect of XIST knockdown on GECs proliferation. (**e**) Effect of XIST knockdown on GECs migration. (**f**) Effect of XIST knockdown on GECs tube formation. (**g**) Effect of XIST knockdown on expression of tight junction-related proteins by western blot assay. (**h**) Effect of XIST knockdown on expression of tight junction-related proteins by immunofluorescence assay. (**i**) Effect of XIST knockdown on expression of CXCR7 by western blot assay. Data represent mean±s.d. (*n*=5, each). *P<0.05 vs XIST(−)NC group, **P<0.01 vs XIST(−)NC group. Scale bar represents 30 μm.

To verify the role of XIST in BTB permeability and glioma angiogenesis, we first evaluated the expression of XIST in GECs and ECs. As shown in Figure 1a, XIST expression in GECs was significantly higher than in ECs. (Supplementary Figure 2A shows the expression of XIST in GECs obtained from co-culturing with U118 cells.) Considering the length of XIST, we knocked down XIST to further investigate its function. Figure 1b shows that transendothelial electric resistance (TEER) decreased in the XIST(−) group compared with the XIST(−) negative control (NC) group, indicating that XIST inhibition impaired BTB integrity. Horseradish peroxidase (HRP) flux was much higher in the XIST(−) group than in the XIST(−)NC group (Figure 1c), indicating that XIST inhibition increased BTB permeability. Figures 1d–f shows that XIST knockdown attenuated the proliferation, migration and tube formation of GECs. These data suggest that XIST knockdown loosens the BTB and impairs human glioma angiogenesis.

### Knockdown of XIST inhibited the expression of tight junction-related proteins and CXCR7 in GECs

ZO-1, ZO-2 and occludin are members of tight junction-related proteins. We focused on these three proteins to investigate the mechanism of action of XIST on BTB permeability. The western blot assays showed that GECs in the XIST(−) group presented lower expression of ZO-1, ZO-2 and occludin compared with the XIST(−)NC group (Figure 1g). Consistent with the western blot results, immunofluorescence confirmed that XIST inhibition suppressed ZO-1, ZO-2 and occludin expression, with discontinuous distribution on the boundaries of GECs (Figure 1h). The western blot analysis also revealed a decrease in CXCR7 expression in the XIST(−) group (Figure 1i), which may explain the way in which XIST knockdown impaired glioma angiogenesis. (Supplementary Figure 3 shows that XIST regulated functions and gene’s expression of GECs that were obtained from co-culturing with U118 cells.)

### XIST bound to miR-137, and XIST and miR-137 were reciprocally repressed

**Figure 2:**
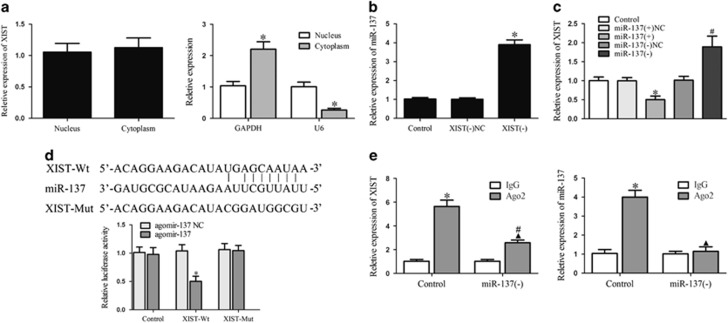
XIST ‘sponged’ miR-137. (**a**) Relative XIST expression in the nucleus and cytoplasm by real-time quantitative PCR (qPCR). GAPDH and U6 were used as cytosol and nucleus markers, respectively. (**b**) Relative miR-137 expression in XIST knockdown GECs by real-time qPCR. Data represent mean±s.d. (*n*=5, each). *P<0.05 vs XIST(−)NC group. (**c**) Relative XIST expression in miR-137 overexpression or knockdown GECs by real-time qPCR. Data represent mean±s.d. (*n*=5, each). *P<0.05 vs miR-137(+)NC group, ^#^*P*<0.05 vs miR-137(−)NC group. (**d**) Relative luciferase activity in HEK293T cells co-transfected pmirGLO-XIST-Wt and agomir-137. Data represent mean±s.d. (*n*=5, each). *P<0.05 vs XIST-Wt+ agomir-137 NC group. (**e**) RNA immunoprecipitation assay was performed with normal mouse IgG or anti-Ago2. Relative expression levels of XIST and miR-137 were determined by quantitative real-time PCR. Data represent mean±s.d. (*n*=5, each). *P<0.01 vs IgG group.

Competing endogenous RNA theory suggests that lncRNA may function as a molecular ‘sponge’ that modulates the biological function of microRNAs (miRNAs).^19, 20^ To confirm competing endogenous RNA theory, the subcellular localization of XIST was first assessed, showing that XIST was localized in both the nucleus and cytoplasm (Figure 2a). The expression of miR-137 was then detected in the XIST-deficient GECs, and the expression of XIST was detected in GECs in which miR-137 was either overexpressed or inhibited. As shown in Figures 2b and c, reciprocal repression between XIST and miR-137 was observed. We next performed a dual-luciferase reporter assay and RNA immunoprecipitation assay to determine whether XIST binds to miR-137. The dual-luciferase reporter assay showed that the co-transfection of pmirGLO-XIST-Wt and agomir-137 resulted in lower luciferase activity compared with the co-transfection of pmirGLO-XIST-Mut and agomir-137 (Figure 2d). The RNA immunoprecipitation assay showed that XIST and miR-137 were enriched in Ago2 immunoprecipitates compared with IgG immunoprecipitates. The downregulation of miR-137 decreased the expression of XIST and miR-137 immunoprecipitated with Ago2 (Figure 2e). These data indicate that XIST regulated miR-137 function by ‘sponging’ it.

### miR-137 was involved in XIST-regulated BTB permeability and glioma angiogenesis

**Figure 3:**
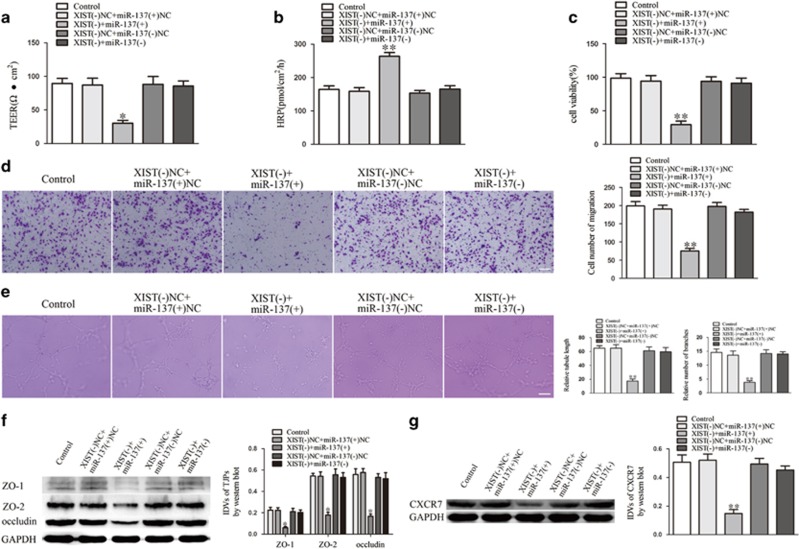
miR-137 mediated the effects of XIST knockdown on GECs. (**a**) TEER assay to evaluate the effect of XIST and miR-137 on BTB integrity. (**b**) HRP flux assay to evaluate the effect of XIST and miR-137 on BTB permeability. (**c**) Cell Counting Kit-8 (CCK-8) assay to evaluate the effect of XIST and miR-137 on GECs proliferation. (**d**) Transwell assay to evaluate the effect of XIST and miR-137 on GECs migration. (**e**) Matrigel tube formation assay to evaluate the effect of XIST and miR-137 on GECs tube formation. (**f**) Western blot assay to evaluate the effect of XIST and miR-137 on tight junction-related proteins. (**g**) Western blot assay to evaluate the effect of XIST and miR-137 on CXCR7. Data represent mean±s.d. (*n*=5, each). *P<0.05 vs XIST(−)NC+miR-137(+)NC group, **P<0.01 vs XIST(−) NC+miR-137(+) NC group. Scale bar represents 30 μm.

Stable XIST-silenced GECs were transfected with antagomir-137 to investigate the effects of miR-137 on XIST-regulated BTB permeability and glioma angiogenesis. As shown in Figures 3a and b, the downregulation of miR-137 largely reversed the XIST inhibition-induced decrease in TEER and increase in HRP flux. The proliferation, migration and tube formation of GECs that were inhibited by XIST knockdown were reversed by miR-137 silencing (Figures 3c–e). Similar to the functional studies, the western blot assays revealed that the lower expression of ZO-1, ZO-2, occludin (Figure 3f) and CXCR7 (Figure 3g) that was induced by XIST knockdown was reversed in the XIST(−)+miR-137(−) group.

### miR-137 regulated BTB permeability, glioma angiogenesis, tight junction-related proteins and CXCR7 expression

**Figure 4:**
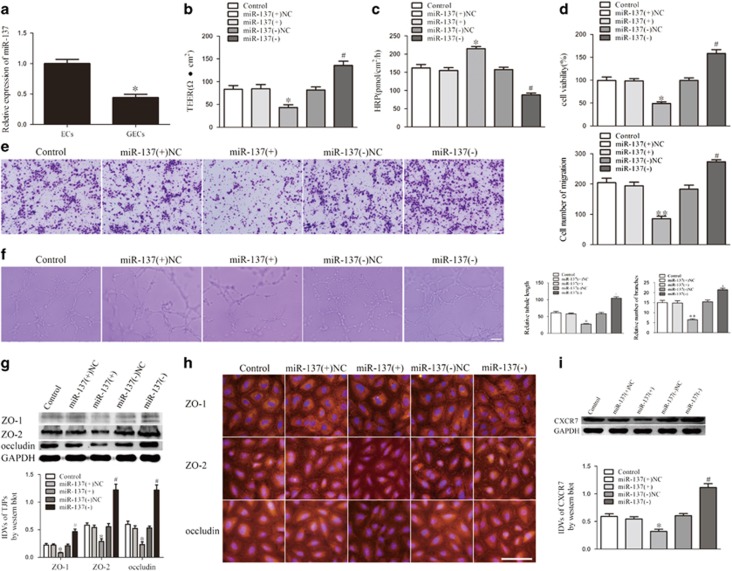
miR-137 expression in GECs and miR-137 regulated BTB permeability, glioma angiogenesis, tight junction-related proteins and CXCR7 expression. (**a**) Relative miR-137 expression in ECs and GECs by real-time quantitative PCR. Data represent mean±s.d. (*n*=5, each). *P<0.05 vs ECs group. (**b**) Effect of miR-137 on TEER in BTB model *in vitro*. (**c**) Effect of miR-137 on HRP flux in BTB model *in vitro*. (**d**) Effect of miR-137 on GECs proliferation. (**e**) Effect of miR-137 on GECs migration. (**f**) Effect of miR-137 on GECs tube formation. (**g**) Effect of miR-137 on expression of tight junction-related proteins by western blot assay. (**h**) Effect of miR-137 on tight junction-related proteins by immunofluorescence assay. (**i**) Effect of miR-137 on expression of CXCR7 by western blot assay. Data represent mean±s.d. (*n*=5, each) *P<0.05 vs miR-137(+)NC group, **P<0.01 vs miR-137(+)NC group, ^#^*P*<0.05 vs miR-137(−)NC group. Scale bar represents 30 μm.

To further evaluate the role of miR-137 in BTB permeability and glioma angiogenesis, we detected the expression of miR-137 in GECs and ECs. miR-137 was downregulated in GECs compared with ECs (Figure 4a). (Supplementary Figure 2B shows the expression of miR-137 in GECs obtained from co-culturing with U118 cells.) The gain- and loss-of-function tests showed that the miR-137(+) group exhibited a decrease in TEER (Figure 4b), and an increase in HRP flux (Figure 4c), compared with the miR-137(+)NC group. Opposite effects were observed in the miR-137(−) group. The upregulation of miR-137 inhibited the proliferation, migration and tube formation of GECs, whereas the downregulation of miR-137 enhanced the proliferation, migration and tube formation of GECs (Figures 4d–f). These results indicate that the ectopic expression of miR-137 impaired BTB integrity, increased BTB permeability and suppressed glioma angiogenesis.

The mechanistic studies showed that ZO-1, ZO-2 and occludin expression was inhibited in the miR-137(+) group, and miR-137 knockdown increased ZO-1, ZO-2 and occludin expression (Figure 4g). Subsequent immunofluorescence showed that ZO-1, ZO-2 and occludin were downregulated in the miR-137(+) group, which exhibited discontinuous distribution on the boundaries of GECs (Figure 4h). CXCR7 was downregulated in the miR-137(+) group compared with the miR-137(+)NC group, and miR-137 inhibition upregulated CXCR7 (Figure 4i). These data indicate that miR-137 may loosen BTB by downregulating ZO-1, ZO-2 and occludin, and inhibit glioma angiogenesis by suppressing CXCR7 expression in GECs.

### miR-137 targeted ZO-2 and FOXC1

**Figure 5:**
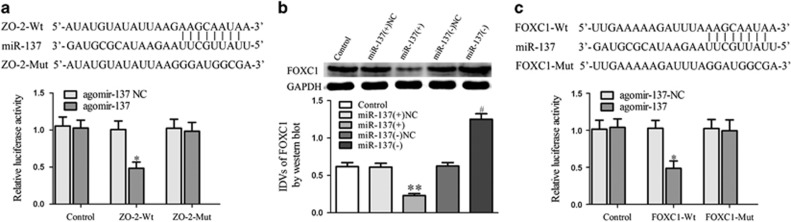
MiR-137 targeted ZO-2 and FOXC1. (**a**) Relative luciferase activity in HEK293T cells co-transfected pmirGLO-ZO-2-Wt and agomir-137. Data represent mean±s.d. (*n*=5, each). *P<0.05 vs ZO-2-Wt+ agomir-137 NC group. (**b**) Effect of miR-137 on expression of FOXC1 by western blot assay. **P<0.01 vs miR-137(+)NC group. ^#^*P*<0.05 vs miR-137(−)NC group. (**c**) Relative luciferase activity in HEK293T cells co-transfected pmirGLO-FOXC1-Wt and agomir-137. Data represent mean±s.d. (*n*=5, each). *P<0.05 vs FOXC1-Wt+agomir-137 NC group.

miRNAs suppress the translation of target messenger RNAs or accelerate their degradation through the RNA-induced silencing complex.^21, 22^ By searching the Targetscan bioinformatics tool, we found that miR-137 only has a putative binding site with the ZO-2 3′-untranslated region, indicating that miR-137 indirectly regulates ZO-1, occludin and CXCR7. Subsequent dual-luciferase reporter assays showed that the co-transfection of pmirGLO-ZO-2-Wt and agomir-137 impaired luciferase activity (Figure 5a). After checking the presumed targets of miR-137, FOXC1 was selected to investigate the precise mechanism of action of miR-137 on BTB permeability and glioma angiogenesis. Western blot revealed that FOXC1 expression was inhibited in the miR-137(+) group (Figure 5b). The dual-luciferase reporter assay revealed that the co-transfection of pmirGLO-FOXC1-Wt and agomir-137 resulted in weaker luciferase activity compared with the co-transfection of pmirGLO-FOXC1-Mut and agomir-137 (Figure 5c).

### FOXC1 was upregulated in GECs, and FOXC1 overexpression decreased BTB permeability by activating ZO-1 and occludin expression and increased glioma angiogenesis by activating CXCR7 expression

**Figure 6:**
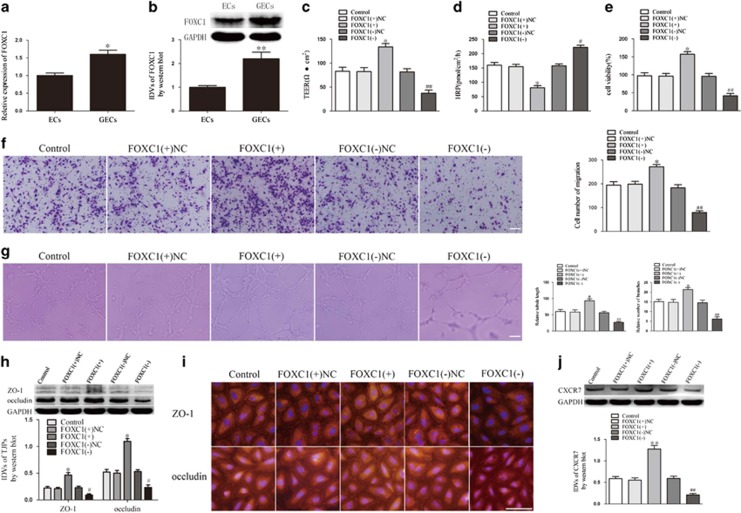
FOXC1 expression in GECs and FOXC1 regulated BTB permeability, glioma angiogenesis, tight junction-related proteins and CXCR7 expression. (**a**) Relative FOXC1 expression in ECs and GECs by real-time quantitative PCR. Data represent mean±s.d. (*n*=5, each). *P<0.05 vs ECs group. (**b**) Relative FOXC1 expression in ECs and GECs by Western blot assay. Data represent mean±s.d. (*n*=5, each). **P<0.01 vs ECs group. (**c**) Effect of FOXC1 on TEER in BTB model *in vitro*. (**d**) Effect of FOXC1 on HRP flux in BTB model *in vitro*. (**e**) Effect of FOXC1 on GECs proliferation. (**f**) Effect of FOXC1 on GECs migration. (**g**) Effect of FOXC1 on GECs tube formation. (**h**) Effect of FOXC1 on ZO-1 and occludin expression by western blot assay. (**i**) Effect of FOXC1 on ZO-1 and occludin by immunofluorescence assay. (**j**) Effect of FOXC1 on CXCR7 expression by western blot assay. Data represent mean±s.d. (*n*=5, each) *P<0.05 vs FOXC1(+)NC group, **P<0.01 vs FOXC1(+)NC group, ^#^*P*<0.05 vs FOXC1(−)NC group, ^##^*P*<0.01 vs FOXC1(−)NC group. Scale bar represents 30 μm.

After confirming that FOXC1 is a downstream target of miR-137, we then detected the expression of FOXC1 in GECs. Real-time quantitative PCR and western blot showed that FOXC1 messenger RNA (Figure 6a) and protein (Figure 6b) were upregulated in GECs. (Supplementary Figures 2C and D show the expression of FOXC1 in GECs obtained from co-culturing with U118 cells.) We next constructed GECs with either FOXC1 overexpression or knockdown to investigate whether FOXC1 regulates BTB permeability and glioma angiogenesis. Compared with the FOXC1(+)NC group, the FOXC1(+) group presented an increase in TEER (Figure 6c) and decrease in HRP flux (Figure 6d). The tests of GECs behavior showed that GECs in the FOXC1(+) group exhibited increases in proliferation, migration and tube formation. The FOXC1(−) group presented the opposite effects (Figures 6e–g). The subsequent western blot assay showed that FOXC1 overexpression increased the expression of ZO-1 and occludin (Figure 6h), and a high fluorescence intensity and cell boundary enrichment of ZO-1 and occludin were observed in GECs in the FOXC1(+) group (Figure 6i). The overexpression of FOXC1 upregulated CXCR7, whereas FOXC1 knockdown downregulated CXCR7 (Figure 6j).

**Figure 7:**
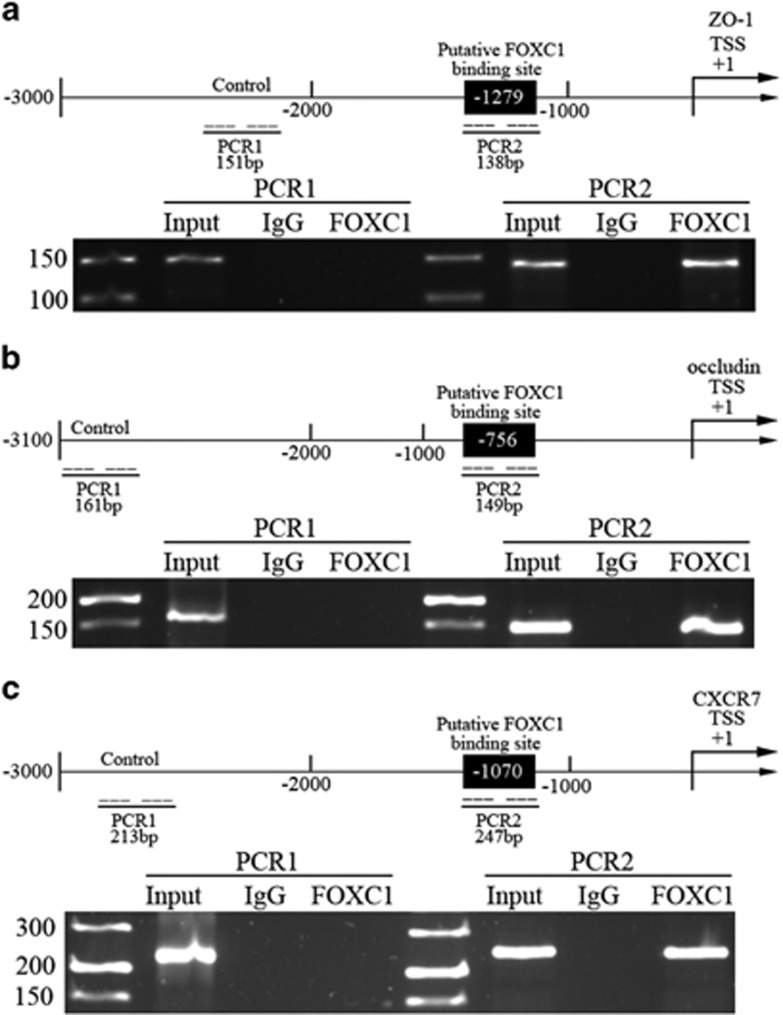
FOXC1 bound to promoter of ZO-1, occludin and CXCR7 in GECs. Schematic representation of the human ZO-1, occludin and CXCR7 promoter regions. Chromatin immunoprecipitation PCR products for putative FOXC1-binding sites and an upstream region not expected to associate with FOXC1 are amplified by PCR using their specific primers PCR of ZO-1 (**a**), occludin (**b**) and CXCR7 (**c**) was conducted with the resulting precipitated DNA.

FOXC1 is reported to bind the 5′-TAAAT/CAA-3′ sequence of target promoters to promote gene transcription.^23^ We searched the promoter regions of ZO-1, occludin and CXCR7, and found that ZO-1 and CXCR7 promoters existed in 5′-TAAATAA-3′ sequence, and the occludin promoter existed in 5′-TAAACAA-3′ sequence. The subsequent chromatin immunoprecipitation assay confirmed that FOXC1 bound to these regions of ZO-1, occludin and CXCR7 in GECs (Figure 7). (Supplementary Figure 4 shows that FOXC1 bound to promoter of ZO-1, occludin and CXCR7 in normal ECs.)

These results indicate that FOXC1 overexpression inhibits BTB permeability by transcriptionally upregulating ZO-1 and occludin, and promotes glioma angiogenesis by transcriptionally upregulating CXCR7.

### FOXC1 regulated miR-137-medicated BTB permeability and glioma angiogenesis, and the expression of ZO-1, occludin and CXCR7

**Figure 8:**
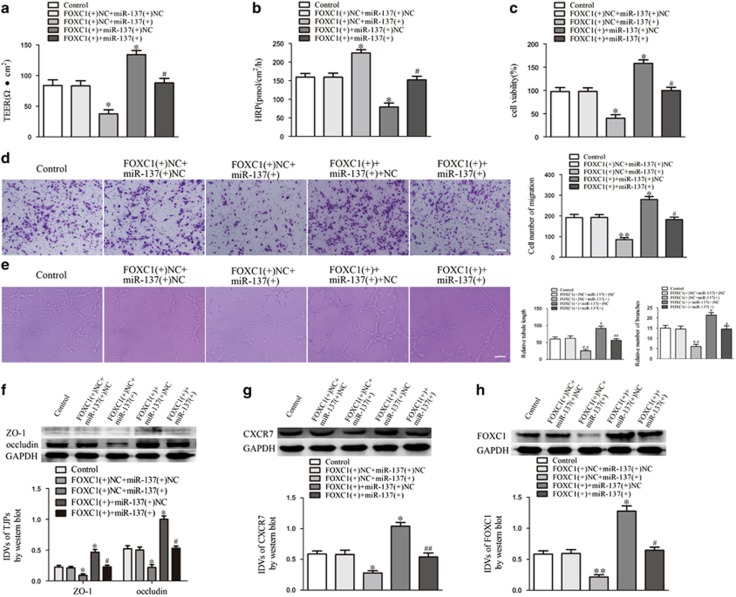
FOXC1 mediated the effects of miR-137 overexpression on GECs. (**a**) TEER assay to evaluate the effect of FOXC1 and miR-137 on BTB integrity. (**b**) HRP flux assay to evaluate the effect of FOXC1 and miR-137 on BTB permeability. (**c**) Cell Counting Kit-8 (CCK-8) assay to evaluate the effect of FOXC1 and miR-137 on GECs proliferation. (**d**) Transwell assay to evaluate the effect of FOXC1 and miR-137 on GECs migration. (**e**) Matrigel tube formation assay to evaluate the effect of FOXC1 and miR-137 on GECs tube formation. (**f**) Western bolt assay to evaluate the effect of FOXC1 and miR-137 on tight junction-related proteins. (**g**) Western bolt assay to evaluate the effect of FOXC1 and miR-137 on CXCR7. Data represent mean±s.d. (*n*=5, each). *P<0.05 vs FOXC1(+)NC+miR-137(+)NC group, **P<0.01 vs FOXC1(+) NC+miR-137(+) NC group. ^#^*P*<0.05 vs FOXC1(+)NC+miR-137(+)group, ^##^*P*<0.01 vs FOXC1(+)NC+miR-137(+) group. Scale bar represents 30 μm.

As shown in Figures 8a and b, the co-overexpression of FOXC1 and miR-137 reversed the effect of miR-137 overexpression alone on TEER and HRP flux. The co-overexpression of FOXC1 and miR-137 also ameliorated the miR-137-regulated inhibition of the proliferation, migration and tube formation of GECs (Figures 8c–e). Western blot assays showed that the co-overexpression of FOXC1 and miR-137 restored ZO-1, occludin, CXCR7 and FOXC1 expression, which were downregulated by miR-137 overexpression alone (Figures 8f–h). These data indicate that FOXC1 is involved in miR-137-regulated BTB permeability and glioma angiogenesis.

## Discussion

In the present study, we found that the lncRNA XIST was upregulated in GECs that were collected in a BTB model *in vitro*. XIST inhibition increased BTB permeability and suppressed glioma angiogenesis. In contrast to XIST, miR-137 was downregulated in GECs, and miR-137 overexpression contributed to the increase in BTB permeability and inhibition of glioma angiogenesis by attenuating FOXC1 and ZO-2 expression. Moreover, FOXC1 presented a negative expression pattern with miR-137 in GECs, which promoted ZO-1 and occludin expression, led to an increase in BTB permeability, and promoted CXCR7 expression that activated glioma angiogenesis.

Tumor ECs play a pivotal role in the BTB.^3^ The behaviors of tumor ECs substantially contributes to glioma angiogenesis.^24^ Therefore, immortalized human brain microvascular ECs were the focus of the present study. We established a BTB model *in vitro* that simulated the glioma microenvironment. TEER and HRP flux were measured to reflect the integrity and permeability of the BTB, respectively. The proliferation, migration and tube formation of GECs reflected glioma angiogenesis.

Emerging evidence shows that lncRNAs regulate the malignant behavior of various tumors.^25, 26, 27^ The aberrant expression of lncRNAs associate with the dysfunction of ECs. LncRNA-RNCR3 has been implicated in atherosclerosis-related EC dysfunction by sponging miR-185-5p.^28^ lncRNA-MIAT is involved in diabetes mellitus-induced retinal pathological angiogenesis, and MIAT was shown to regulate the behavior of ECs that were cultured in a high-glucose medium.^29^ The present study provided evidence that lncRNA XIST regulates GECs function. XIST inhibition increased BTB permeability by decreasing ZO-1, ZO-2 and occludin expression in GECs. Most lipophilic agents are transported paracellularly, whereas hydrophilic agents are delivered through transcellular pathway.^30^ The caveolae-mediated transcellular pathway plays an important role in increasing BTB permeability.^31, 32^ The present study focused on the paracellular pathway. Whether XIST knockdown increases BTB permeability through the caveolae-mediated transcellular pathway deserves further investigation. XIST inhibition also impaired GECs proliferation, migration and tube formation, which are the main processes associated with glioma angiogenesis. The mechanism of tumor angiogenesis that is mediated by lncRNAs is complicated,^33, 34, 35^ and the vascular endothelial growth factor A pathway plays a crucial role.^36, 37^ We found that XIST inhibition suppressed glioma angiogenesis by downregulating the expression of CXCR7. Whether vascular endothelial growth factor A or other mediators of angiogenesis are involved in XIST-regulated glioma angiogenesis also deserves further investigation.

Our search of starbase 2.0 (http://starbase.sysu.edu.cn/) revealed that miR-137 is a presumed target of XIST. Thus, we hypothesized that XIST regulates BTB permeability and glioma angiogenesis by sponging miR-137. Further analyses showed that miR-137 expression in GECs was negatively correlated with XIST expression, and XIST knockdown significantly upregulated miR-137. Overexpression of miR-137 reversed the influence of XIST deficiency on BTB permeability and glioma angiogenesis. Subsequent RNA immunoprecipitation and luciferase assays confirmed that miR-137 was enriched by XIST and bound to XIST in a sequence-specific manner, respectively. These results support our hypothesis that XIST functions as a ‘sponge’ to regulate miR-137. Consistent with our results, previous studies have shown that XIST functions as a miRNA ‘sponge’. XIST has been reported to sponge miR-34a-5p in nasopharyngeal carcinoma^38^ and sponge miR-101 in gastric cancer.^39^ In addition to ‘sponge’ miRNAs, lncRNAs also epigenetically regulate miRNAs. LncRNA-GIHCG contributes to histone H3K27 trimethylation and DNA methylation of the miR-200b/a/429 promoter.^40^ NEAT1 promotes the DNA methylation of the CpG island in the miR-129 gene.^41^ Both GIHCG and NEAT1 epigenetically silence target miRNA expression.^40, 41^ The miR-137 promoter has been shown to be hypermethylated in several tumor samples and cell lines, including lung cancer,^42^ colorectal cancer^43^ and GBM.^44^ An interesting line of investigation would be to evaluate whether XIST epigenetically regulates miR-137.

miR-137 has been proposed to exert a tumor-suppressing effect in various tumors, such as colon cancer,^45^ multiple myeloma^46^ and glioma,^9, 10^ among others. Moreover, miR-137 inhibition ameliorates the high-glucose-induced decrease in cell viability and increases human umbilical vein endothelial cells apoptosis.^47^ In the present study, miR-137 overexpression increased BTB permeability by downregulating ZO-1, ZO-2 and occludin in GECs. The overexpression of miR-137 inhibited GECs proliferation, migration and tube formation by downregulating CXCR7. Consistent with our results, a recent study found that the ectopic expression of miR-137 in glioma cell lines significantly inhibits glioma-driven angiogenesis.^12^ miRNAs bind to the 3′-untranslated region of target messenger RNA to inhibit gene expression posttranscriptionally.^21^ DCLK1,^45^ SP1^48^ and CUL4A^49^ are confirmed targets of miR-137. In the present study, we utilized bioinformatics tools and performed a dual-luciferase assay and found that ZO-2 is a novel target of miR-137, suggesting that miR-137 indirectly regulates ZO-1, occludin and CXCR7.

To elucidate the mechanism of action of miR-137 on BTB permeability and glioma angiogenesis, we confirmed that FOXC1 is a target of miR-137. FOXC1 is a member of forkhead box transcription factors family. The silencing of FOXO3A inhibits hypoxia-induced brain–blood barrier hyperpermeability,^50^ and FOXM1B activates glioma angiogenesis by promoting vascular endothelial growth factor expression.^51^ FOXC1 plays an oncogenic role in melanoma^52^ and endometrial carcinoma,^53^ and has been reported to regulate the function and gene expression of ECs. The overexpression of FOXC1 increases human umbilical vein endothelial cells migration.^54^ FOXC1 activates the Hey2 promoter and promotes the Hey2 transcription in human umbilical vein endothelial cells.^55^ In the present study, we found that FOXC1 was upregulated in GECs, and FOXC1 overexpression decreased BTB permeability by upregulating ZO-1 and occludin in GECs. In addition, FOXC1 increased the proliferation, migration and tube formation of GECs. However, Fatima^56^ showed that FOXC1 silencing increases mouse lymphatic ECs proliferation through the hyperactivation of extracellular signal-regulated kinase. These disparate results may be attributable to the different origin and species of ECs in the two studies.

CXCR7 is a CXCL12 receptor and important modulator of tumor angiogenesis.^57, 58^ CXCR7 knockdown in hepatocellular carcinoma cells inhibits tumor angiogenesis,^58^ and CXCR7 overexpression promotes tumor angiogenesis by activating the AKT pathway.^59^ Moreover, CXCR7 is correlated with papillary thyroid carcinoma angiogenesis, likely by affecting interleukin-8 and vascular endothelial growth factor expression.^57^ CXCR7 inhibition was shown to suppress human brain microvessel ECs proliferation, migration and tube formation.^60^ CXCR7 also plays a more significant regulatory role in tumor ECs compared with normal ECs.^61^ Takano^62^ showed that ECs that were isolated from GBM patients had high levels of CXCR7 messenger RNA. In the present study, we found that FOXC1 upregulation promoted CXCR7 expression by activating the CXCR7 promoter, indicating that CXCR7 is involved in FOXC1-regulated glioma angiogenesis.

In conclusion, the present study found that XIST and FOXC1 expression increased and miR-137 expression decreased in GECs. XIST knockdown increased BTB permeability and inhibited glioma angiogenesis by inhibiting FOXC1 and ZO-2 expression through upregulation of miR-137. These findings may contribute to the development of effective strategies for the treatment of glioma.

## Materials and methods

### Cell lines and cultures

The immortalized human brain EC line hCMEC/D3 was gifted from Dr Couraud (Institut Cochin, Paris, France). ECs were cultured as described previously.^63^ ECs applied in this study were limited from 30 to 35 passages. Human glioma cell lines U87MG, U118MG and human embryonic kidney 293T (HEK293T) cell line were purchased from the Shanghai Institutes for Biological Sciences Cell Resource Center and were cultured in Dulbecco’s modified Eagle medium of high glucose containing 10% fetal bovine serum. All cells were maintained in a humidified incubator (37 °C, 5% CO_2_).

### Establishment of an *in vitro* BTB model

The *in vitro* BTB model was established as described previously.^63^ Briefly, U87 cells were seeded in six-well plates at 2 × 10^4^ per well. After culturing U87 cells for 2 days, ECs were seeded on the upper side of inserts coated with Cultrex Rat Collagen I (R&D Systems, Minneapolis, MN, USA) at a density of 2 × 10^5^ per well. Both U87 cells and ECs were maintained with prepared EBM-2 (endothelial basal medium 2), and medium was refreshed every 2 days. GECs were obtained after co-culturing with U87 cells for 4 days.

### Real-time PCR assay

Total RNA was extracted from cells by using Trizol reagent (Invitrogen, Carlsbad, CA, USA), according to the manufacturer’s protocol. The nuclear and cytoplasmic fractions were separated by PARIS Kit (Life Technologies, Foster City, CA, USA), as described by the manufacturer. SYBR Premix Ex Taq and TaqMan gene expression assays (Applied Biosystems, Foster City, CA, USA) were used for the detections of XIST, FOXC1, U6 and GAPDH. TaqMan MicroRNA Reverse Transcription kit and Taqman Universal Master Mix II (Applied Biosystems) were used to detect miR-137 and U6 expression. Relative expression values were normalized and calculated using the relative quantification (2^−△△Ct^) method. Primers and probes used for quantitative PCR with reverse transcription were shown in Supplementary Table 1.

### Cell transfection

The short-hairpin RNA direct against human XIST (NR_001564.2) gene or FOXC1 (NM_001453.2) gene was reconstructed in a pGPU6/GFP/Neo vector (XIST(−), FOXC1(−); GenePharma, Shanghai, China), respectively. Its empty vector was used as a NC (XIST(−)NC, FOXC1(−)NC). Human FOXC1 gene coding sequence was ligated into pIRES2-EGFP vector (FOXC1(+)) (GeneScript, Piscataway, NJ, USA), and its empty vector was used as a NC (FOXC1(+)NC).

ECs were seeded in 24-well plates and transfected using LTX and Plus reagent (Life Technologies) when the confluence reached at ~80%. Stable cell lines were selected through the medium containing Geneticin (G418; Sigma-Aldrich, St Louis, MO, USA), G418-resistant clones were obtained after 4 weeks.

Agomir-137 (miR-137(+)), antagomir-137 (miR-137(−)) and their NC sequence (miR-137(+)NC and miR-137(−)NC; GenePharma) were transiently transfected into ECs, ECs which stably transfected shXIST or FOXC1 overexpression, respectively, according to the manufacturer’s instructions using lipofectamine 3000 reagent. Cells were collected 48 h after transfection.

Sequences of shXIST, shFOXC1 and shNC were shown in Supplementary Table 2. The transfection efficiency of XIST, FOXC1 and miR-137 was shown in Supplementary Figure 1.

### TEER assays

TEER assay was performed in the *in vitro* BTB model using millicell-ERS instrument (Millipore, Billerica, MA, USA). Co-culture inserts were placed in room temperature for 30 min before TEER assay was performed. TEER value was measured immediately after the culture medium was renewed. Background electrical resistance was subtracted before calculating the final resistances (Ω cm^2^).

### HRP flux assays

The permeability of the *in vitro* BTB was determined by detecting HRP (44 kDa) permeability. After the BTB model was established, 1 ml of serum-free EBM-2 medium containing 10 μg/ml HRP was added to the upper compartment of inserts and 2 ml of complete medium was added to the well. Then, 5 μl of culture medium was gathered from the lower compartment after incubated at 37 °C for 1 h. Samples were detected by tetramethylbenzidine colorimetry approach. Absorbance at 370 nm was measured with a spectrophotometer. The final HRP flux was expressed as pmol passed per cm^2^ surface area per hour.

### Cell proliferation assay

Cells were seeded at the density of 2000 cells peer well in 96-well plates. According to the manufacturer’s instructions, 10 μl of CCK-8 (Beyotime Institute of Biotechnology, Jiangsu, China) was added into each well. After incubated at 37 °C for 2 hours, absorbance at 450 nm was recorded.

### Cell migration assay

Cells were resuspended in 200 μl serum-free medium at a density of 2 × 10^5^ cells per ml and added to the upper chamber of a 24-well transwell chamber (8 μm pore size, Corning, Corning City, NY, USA), whereas the lower chamber was added to 600 μl of EBM-2 medium supplemented with 5% fetal bovine serum. The transwell system was incubated for 24 h at 37 °C. Then, cells on the upper surface of the membrane were removed with cotton swabs. Cells on the lower surface of the membrane were fixed with methanol and glacial acetic acid at the ratio of 3:1 and strained with 10% Giemsa. Then, stained cells of five vision fields were randomly chosen for statistics.

### Tube formation assay

A volume of 100 μl Matrigel (BD Biosciences, Bedford, MA, USA) was added to pre-cooled 96-well plates and incubated at 37 °C for 30 min. Then, cells were resuspended in 100 μl complete EBM-2 medium at a density of 4 × 10^5^ cells per ml and added to Matrigel-coated wells. After maintained in 37 °C for 24 h, photos were collected using Olympus DP71 immunofluorescence microscopy (Olympus, Tokyo, Japan). Total tubule length and number of tubule branches were measured using Chemi Imager 5500 V2.03 software (Alpha Innotech, San Leondro, CA, USA).

### Western blot assays

Equal amounts of protein samples went electrophoresis in SDS–polyacrylamide gel electrophoresis and then transferred to polyvinylidene fluoride membranes. Membranes were blocked to avoid non-specific bindings. Then, primary antibodies (ZO-1, 1:300, Life Technologies Corp., 61-7300; ZO-2, 1:1000, Life Technologies Corp., 38-9100; occludin, 1:600, Abcam, Cambridge, UK, ab31721; CXCR7, 1:1000, Abcam, ab38089; FOXC1, 1:500, Abcam, ab5079; GAPDH, 1:1000, Santa Cruz Biotechnology, Santa Cruz, CA, USA, sc-32233) were incubated with the membranes at 4 °C overnight. Membranes were washed and then incubated with secondary antibodies at room temperature for 2 h. After washing, immune complexes were visualized by enhanced chemiluminescence and scanned using the Chemi Imager 5500 V2.03 software. The integrated density values were calculated using FluorChem 2.0 software (Alpha Innotech).

### Immunofluorescence assays

Cells on insert filters were fixed with 4% paraformaldehyde for 20 min and permeated with 0.3% Triton X-100 for 2 min at room temperature (ZO-1 and ZO-2), or fixed with methanol for 10 min at −20 °C (occludin). Then, cells were blocked in 5% in bovine serum album in phosphate-buffered saline for 2 h at room temperature and incubated with primary antibodies for ZO-1 (1:50; Life Technologies Corp., 61-7300), ZO-2 (1:50; Life Technologies Corp., 38-9100) and occludin (1:50; Abcam, ab31721) at 4 °C overnight. The NC group was incubated with 1% bovine serum album. After three washes with phosphate-buffered saline, cells were incubated with Alexa Fluor 555-labeled goat anti-rabbit IgG secondary antibody (1:500; Beyotime Institute of Biotechnology, Hangzhou, Jiangsu, China) for 2 h at room temperature. 4,6-Diamidino-2-phenylindole was added to the samples to visualize cell nuclei. The staining was analyzed using Olympus DP71 immunofluorescence microscopy and merged with Chemi Imager 5500 V2.03 software.

### Reporter vector construction and luciferase reporter assay

The potential binding sequence of miR-137 in XIST gene and its mutant sequence was amplified by PCR, synthesized and cloned into the pmirGLO dual-luciferase vector (Promega, Madison, WI, USA). Wild-type pmirGLO-XIST (or XIST mutant) reporter plasmid and agomir-137 or agomir-137-NC were co-transfected into HEK293T cells. Luciferase activity was measured 48 h after transfection through the Dual-Luciferase Reporter System (Promega). The 3′-untranslated regions of ZO-2 or FOXC1 containing the presumed miR-137 binding sequences and their mutant sequence were cloned into dual-luciferase vectors. Following transfection approach and measurement of luciferase activities were performed as described above.

### RNA immunoprecipitation assay

Magna RNA-binding protein immunoprecipitation kit (Millipore) were used to perform RNA immunoprecipitation assay. Briefly, cells were lysed in complete RNA lysis buffer and cell lysate were incubated with human anti- Ago2 antibody (Millipore) and NC mouse IgG (Millipore). Samples were incubated with Proteinase K buffer and then immunoprecipitated RNA was isolated. Purified RNA was obtained and then applied to quantitative PCR with reverse transcription analysis.

### Chromatin immunoprecipitation assay

Chromatin immunoprecipitation assay was performed using the Simple Chip Enzymatic Chromatin IP kit (Cell Signaling Technology, Danvers, MA, USA) according to the manufacturer’s instructions. Briefly, cells were crosslinked with EBM-2 containing 1% formaldehyde for 10 min and added glycine for 5 min at room temperature to quenched crosslink. Then, cells were collected in lysis buffer containing 1% phenylmethanesulfonyl fluoride (PMSF). Chromatin was digested by micrococcal nuclease. Lysate (2%) was used as an input reference. Immunoprecipitation was incubated with 3 μg of anti-FOXC1 antibody (Abcam, ab5079) or normal rabbit IgG followed by immunoprecipitating with Protein G Agarose Beads during an overnight incubation at 4 °C with gentle shaking. The DNA crosslink was reversed by 5 mol/l NaCl and Proteinase K at 65 °C for 2 h and then DNA was purified. Immunoprecipitated DNA was amplified by PCR using their specific primers. Primers used for chromatin immunoprecipitation PCR were shown in Supplementary Table 3.

### Statistical analysis

Data were presented as means±s.d. from at least three independent experiments. GraphPad Prism v5.01 (GraphPad Software, La Jolla, CA, USA) software with the Student’s *t*-test (two-tailed) was used for statistical analysis. Differences were considered to be statistically significant when probability *P*<0.05.
