# Supplementary material for: Knockdown of long non-coding RNA XIST increases blood–tumor barrier permeability and inhibits glioma angiogenesis by targeting miR-137
Source: Oncogenesis. 2017 Mar 13;6(3):e303–. doi: 10.1038/oncsis.2017.7 (PMC5533948; doi:10.1038/oncsis.2017.7)
Supplement: Supplementary Figures [file oncsis20177x1.docx]

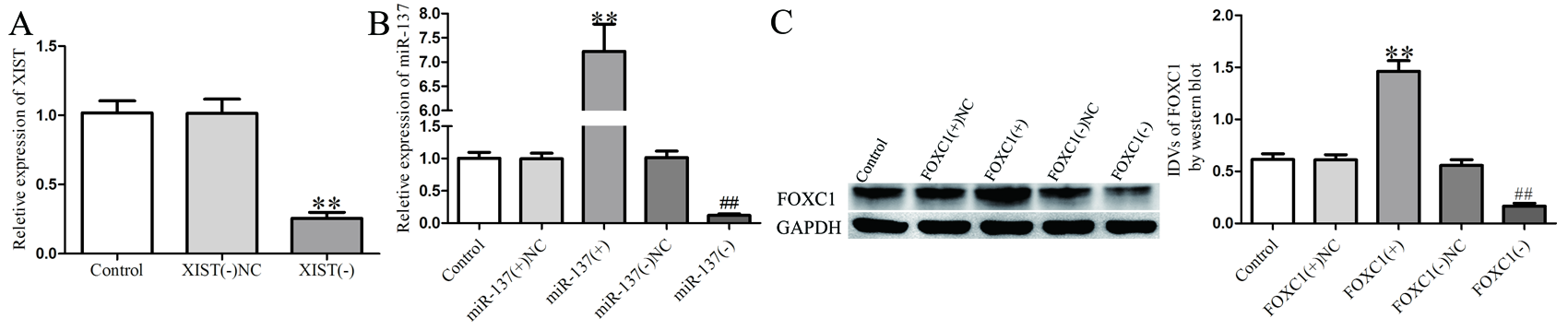


**Fig. 1 Tansfection efficiency of XIST, FOXC1 and miR-137.** A. Relative expression of XIST in cells transfected shXIST. Data represent mean ± SD (n=5, each). ^**^*P*<0.01 vs. XIST(-)NC group. B. Relative expression of miR-137 in cells transfected agomir-137 or antagomir-137. Data represent mean ± SD (n=5, each). ^**^*P*<0.01 vs. miR-137(+)NC group, ^##^*P*<0.01 vs. miR-137(-)NC group. C. Relative expression of FOXC1 in cells transfected pIRES2-EGFP-FOXC1-CDS or shFOXC1. Data represent mean ± SD (n=5, each). ^**^*P*<0.01 vs. FOXC1(+)NC group, ^##^*P*<0.01 vs. FOXC1(-)NC group.


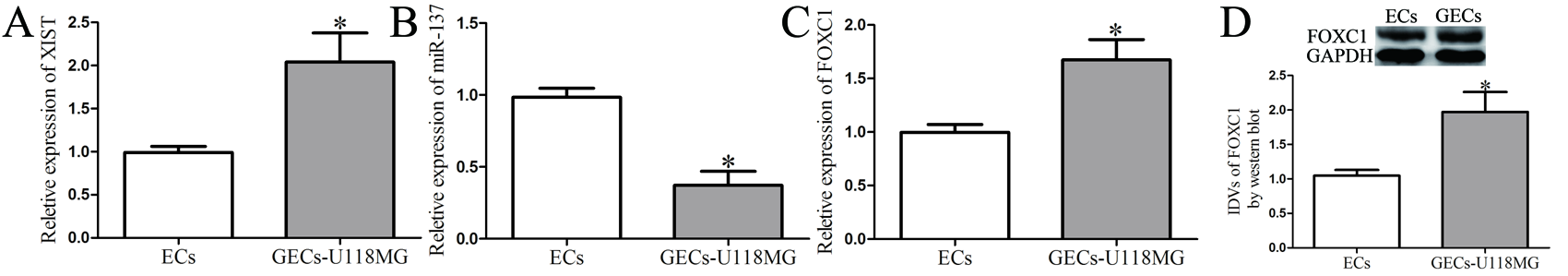


**Fig. 2** **Relative expression of XIST, miR-137 and FOXC1 in EC and GECs obtained from co-culturing with U118-MG**. A. Relative XIST expression in ECs and GECs by Real-time qPCR. B. Relative miR-137 expression in ECs and GECs by Real-time qPCR. C. Relative FOXC1 mRNA expression in ECs and GECs by Real-time qPCR. D. Relative FOXC1 protein expression in ECs and GECs by Western blot. Data represent mean ± SD (n=5, each). **P*<0.05 vs. ECs group.


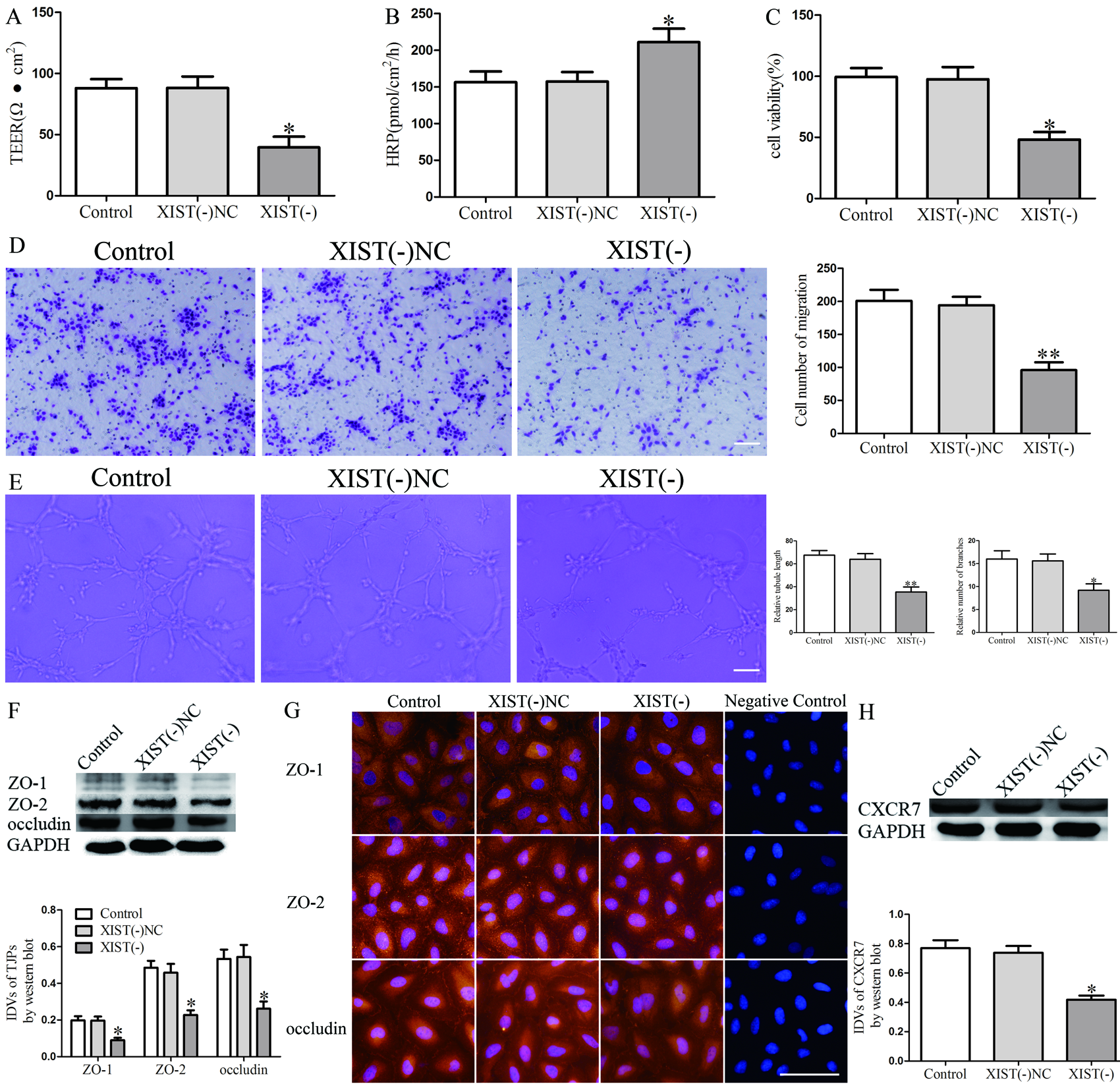


**Fig. 3** XIST regulated functions and genes expression in GECs that were obtained from co-culturing with U118-MG. A. Effect of XIST knockdown on TEER in BTB *in vitro*. B. Effect of XIST knockdown on HRP flux in BTB model *in vitro*. C. Effect of XIST knockdown on GECs proliferation. D. Effect of XIST knockdown on GECs migration. E. Effect of XIST knockdown on GECs tube formation. F. Effect of XIST knockdown on expression of tight junction-related proteins by Western blot assay. G. Effect of XIST knockdown on expression of tight junction-related proteins by immunofluorescence assay. H. Effect of XIST knockdown on expression of CXCR7 by Western blot assay. Data represent mean ± SD (n=5, each) **P*<0.05 vs. XIST(-)NC group, ***P*<0.01 vs. XIST(-)NC group. Scale bar represents 30 μm.


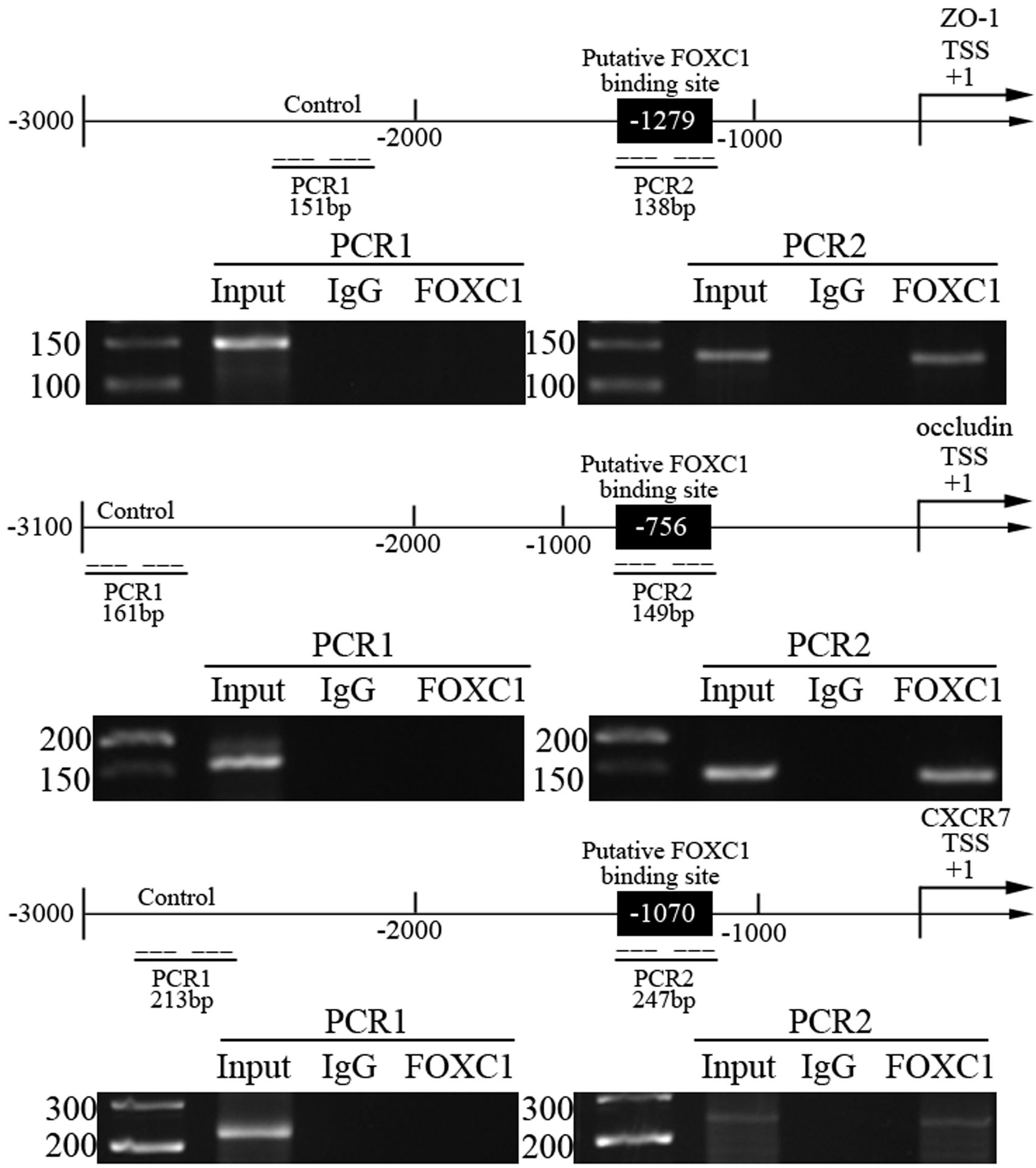


**Fig. 4 FOXC1 bound to promoter of ZO-1, occludin and CXCR7 in normal ECs**. Schematic representation of the human ZO-1, occludin, and CXCR7 promoter regions. ChIP PCR products for putative FOXC1 binding sites and an upstream region not expected to associate with FOXC1 are amplified by PCR using their specific primers PCR of ZO-1 (A), occludin (B) and CXCR7 (C) was conducted with the resulting precipitated DNA.


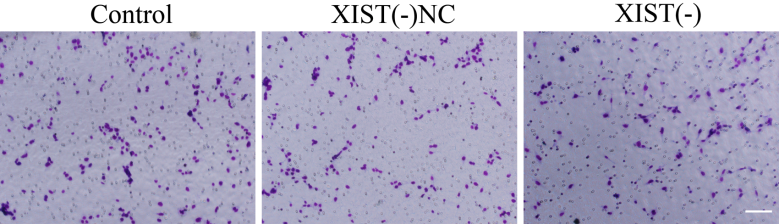

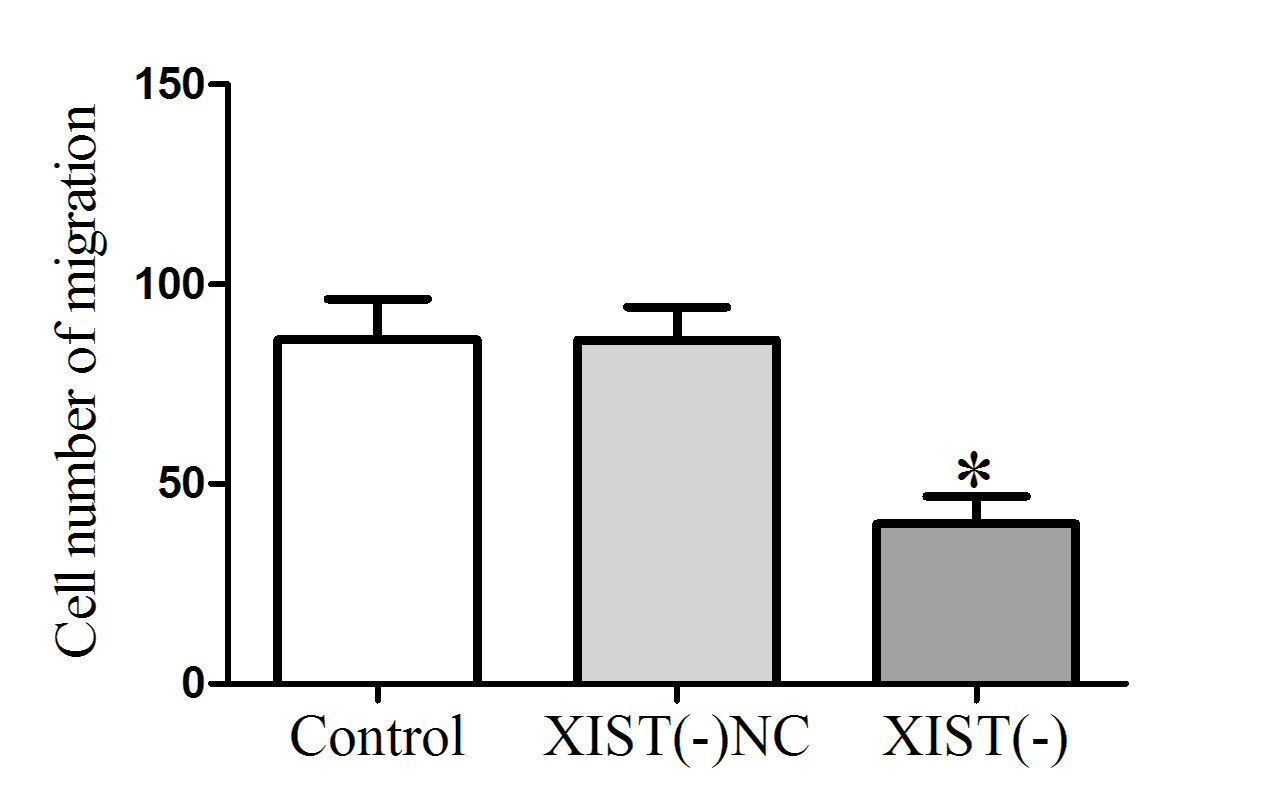


Fig. 5 XIST regulated GECs migration. Effect of XIST knockdown on GECs migration, which were incubated in Transwell system for 6 hour. Data represent mean ± SD (n=5, each) **P*<0.05 vs. XIST(-)NC group.
