# Supplementary material for: Knockdown of long non-coding RNA XIST increases blood–tumor barrier permeability and inhibits glioma angiogenesis by targeting miR-137
Source: Oncogenesis. 2017 Mar 13;6(3):e303–. doi: 10.1038/oncsis.2017.7 (PMC5533948; doi:10.1038/oncsis.2017.7)
Supplement: Supplementary Tables [file oncsis20177x2.docx]

| Primer or Probe | Gene | Sequence (5'->3') or Assay ID |
| --- | --- | --- |
| Primer | XIST | F: AGGTCAGGCAGAGGAAGTCA |
|  |  | R: CTCCCGATACAACAATCACG |
|  | FOXC1 | F: CGGCTTGAACAACTCTCCAG |
|  |  | R: ACAGTCGTAGACGAAAGCTCC |
|  | GAPDH | F: ACAGTCAGCCGCATCTTCTT |
|  |  | R: GCCCAATACGACCAAATCC |
|  | U6 | F:CTCGCTTCGGCAGCACA  R: AACGCTTCACGAATTTGCGT |
| Probe | MiR-137 | 000593(Applied biosystems) |
|  | U6 | 001973(Applied biosystems) |

Table 1. Primers and probes used for RT-qPCR.

Table 2. Sequences of shRNA template

| Gene |  | Sequence(5'->3') |
| --- | --- | --- |
| XIST | Sence | CACCGCTCTTGAACAGTTAATTTGCTTCAAGAGAGCAAATTAACTGTTCAAGAGCTTTTTG |
|  | Antisence | GATCCAAAAAAGCTCTTGAACAGTTAATTTGCTCTCTTGAAGCAAATTAACTGTTCAAGAGC |
| FOXC1 | Sence | CACCGGAGCTTTCGTCTACGACTGTTTCAAGAGAACAGTCGTAGACGAAAGCTCCTTTTTTG |
|  | Antisence | GATCCAAAAAAGGAGCTTTCGTCTACGACTGTTCTCTTGAAACAGTCGTAGACGAAAGCTCC |
| NC | Sence | CACCGTTCTCCGAACGTGTCACGTTTCAAGAGAACGTGACACGTTCGGAGAATTTTTTG |
|  | Antisence | GATCCAAAAAATTCTCCGAACGTGTCACGTTCTCTTGAAACGTGACACGTTCGGAGAAC |

Table 3. Primers used for ChIP experiments

| Gene | Binding site or Control | Sequence (5'->3') | Product size (bp) | Annealing temperature (°C) |
| --- | --- | --- | --- | --- |
| ZO-1 | PCR1 | F: GGAAAAGTGAAAAATGTCAGTGC | 151 | 54 |
|  |  | R: AAAAGGTGGTGATGAAAGACC |  |  |
|  | PCR2 | F: GCCTGGCCCACAATTCTTA | 138 | 53 |
|  |  | R: GCCTTCTGCAAACCAAACC |  |  |
| occludin | PCR1 | F: GAGACTTGTTTCGTGGCTCA | 161 | 53 |
|  |  | R: GGTAGCTGGGTAGGACTAAAGG |  |  |
|  | PCR2 | F: ACCCGGAACTAAGGTGCTTT | 149 | 53 |
|  |  | R: ATTATGGCTCTTTGGCCTGA |  |  |
| CXCR7 | PCR1 | F: CAATCCCGGCTCACTACAAC | 213 | 55 |
|  |  | R: GGCCTGTAATCCCAGCACT |  |  |
|  | PCR2 | F: GAGATGTCCTTGGTGCCGAT | 247 | 55 |
|  |  | R: ACGTGGCTAGAAAGTCTCCG |  |  |
